# Accuracy of low-field magnetic resonance imaging for differentiating intervertebral disc extrusions and protrusions at the lumbosacral disc space in dogs

**DOI:** 10.3389/fvets.2023.1279378

**Published:** 2023-11-06

**Authors:** Hannah Shing, Abby Caine, Giunio Bruto Cherubini, Tim Sparks, Daniel Housley

**Affiliations:** ^1^Dick White Referrals, Part of Linnaeus Veterinary Limited, Cambridgeshire, United Kingdom; ^2^Department of Veterinary Sciences, University of Pisa, Pisa, Tuscany, Italy; ^3^Waltham Petcare Science Institute, Leicestershire, United Kingdom; ^4^VetCT, Cambridgeshire, United Kingdom

**Keywords:** magnetic resonance imaging, intervertebral disc extrusion, intervertebral disc protrusion, lumbosacral, dog

## Abstract

**Introduction/Purpose:**

MRI features differentiating extrusion from protrusion in thoracolumbar discs have been published, however little specifically evaluates the lumbosacral disc. The high prevalence of degenerative changes in apparently normal animals complicates assessment of this region and features relevant elsewhere in the spine may not apply. The aims of this study were to determine the accuracy of MRI in differentiating IVDE and IVDP at the lumbosacral disc space in dogs and determine which MRI characteristics discriminate between IVDE and IVDP.

**Method:**

MRI examinations from dogs with surgically confirmed IVDE or IVDP at the lumbosacral disc space were collected retrospectively (2011–2019). Two radiologists independently recorded a diagnosis of IVDE or IVDP, gave a confidence rating, and evaluated specific MRI features. Univariable statistical analysis was performed to identify which MRI characteristics might help distinguish IVDE from IVDP.

**Results:**

117 dogs with lumbosacral IVDE (*n* = 16) or IVDP (*n* = 101) were included. Features associated with IVDE were in concordance with previous studies and included interruption of the dorsal annulus, suspected epidural hemorrhage, dispersed (rather than confined) intervertebral disc herniation on T2W sagittal images, lateralized intervertebral disc herniation and displacement of the cauda equina. Overall diagnostic accuracy was 68.8% and interobserver agreement was fair (κ = 0.37), which is lower than has been reported in thoracolumbar disc herniation, but accuracy increased to 85.3% with substantially improved agreement (κ = 0.87) in “confident” diagnoses.

**Discussion/Conclusion:**

MRI characteristics used in differentiating thoracolumbar IVDE and IVDP can be extrapolated to the lumbosacral intervertebral disc space, but diagnostic accuracy in low-field MRI is lower than previously reported in herniations involving the thoracolumbar spine.

## Introduction

Herniation of the lumbosacral intervertebral disc, most commonly protrusion, is one component of the multifactorial etiology of degenerative lumbosacral stenosis (1, 2). The pathogenesis and clinical characteristics of intervertebral disc extrusion and intervertebral disc protrusion are different (3). Intervertebral disc extrusion occurs when there is displacement of the nucleus pulposus through a tear in the annulus fibrosus into the surrounding soft tissue, whereas intervertebral disc protrusion is defined as the partial displacement of the nucleus pulposus into a disrupted annulus fibrosus due to rupture of the inner layers of the annulus, and secondary annular hypertrophy (3). Differentiating lumbosacral intervertebral disc extrusion from protrusion may be important for patient treatment and management and carry prognostic implications (4). This differentiation could affect the suitability of non-surgical therapy, or the type of surgical therapy such as dorsal laminectomy with or without discectomy or fixation-fusion (5). Previous studies have found that intervertebral disc protrusion at the lumbosacral space is associated with degenerative changes to the lumbosacral disc and space, and suggested that additional stabilization techniques may be indicated (4–6). Intervertebral disc extrusion at the lumbosacral space, unlike protrusion, may not be associated with chronic instability and therefore have a better prognosis after surgery than dogs with intervertebral disc protrusion and degenerative lumbosacral stenosis (5, 7). It is therefore important to accurately differentiate intervertebral disc extrusions and protrusions at the lumbosacral space, however there are limited data regarding the accuracy of magnetic resonance imaging (MRI) in distinguishing extrusion from protrusion specifically at this site.

The high prevalence of degenerative changes at the lumbosacral space in apparently normal older animals (8–10) makes it difficult to diagnose clinically relevant intervertebral disc herniation and may make it difficult to differentiate extrusion and protrusion. Although guidelines to aid in the differentiation of intervertebral disc extrusion and protrusion have been established for thoracolumbar degenerative disc diseases (4, 6) and the MRI appearance of lumbosacral disc protrusion has been widely described, to the authors’ knowledge only one case series describes the MRI appearance of the less frequently encountered lumbosacral disc extrusion (11). Furthermore, previous studies have also found the agreement between MRI findings of degenerative lumbosacral disc protrusion and surgical findings to be only slight to fair (7, 12).

The aim of this study was therefore to determine the accuracy of MRI for differentiating intervertebral disc extrusion and protrusion at the lumbosacral disc space in dogs in radiologists blinded to all patient data other than the MRI images. A second objective was to determine whether previously reported MRI characteristics used to differentiate protrusion from extrusion remained relevant at the lumbosacral disc space. It was hypothesized that the diagnostic accuracy of MRI in differentiating intervertebral disc extrusion from protrusion at the lumbosacral space would be poor. However, we speculated that specific MRI characteristics would independently aid in differentiating intervertebral disc extrusion from protrusion.

## Materials and methods

### Sample population

This was a retrospective cross-sectional study investigating the accuracy of MRI in diagnosing intervertebral disc extrusions and intervertebral disc protrusions at the lumbosacral disc space. The digital medical database from Dick White Referrals was searched between January 2011 and August 2019 for dogs that had undergone MRI and decompressive surgery for intervertebral disc herniation at the lumbosacral disc space. Images were acquired using a 0.4 Tesla permanent-magnet MRI scanner (Aperto Lucent, Hitachi Medical Corporation, Tokyo, Japan). All patients were scanned under general anesthesia with different anesthetic protocols for premedication and induction depending on the assessment of the attending anesthetist, and isoflurane for maintenance of anesthesia. The dogs were positioned in dorsal recumbency with the lumbosacral spine positioned inside a human knee or open-head coil. Cases were included if (1) T2-weighted (T2W) sagittal and T2W transverse MRI images were available (slice thickness: 3–4 mm, repetition time: TR = 100–120 ms, TE = >2000 ms), (2) following a diagnosis of intervertebral disc herniation they underwent spinal surgery, and (3) the surgical confirmation of type of intervertebral disc herniation (extrusion or protrusion) was clearly recorded. A single reviewer recorded the following from the medical record: patient signalment (breed, age, sex) and surgical diagnosis. Dogs were excluded if the imaging studies were incomplete (minimum availability of a T2W sagittal and T2W transverse series), the type of herniation (extrusion or protrusion) was not clearly recorded, or if more than one type of intervertebral disc herniation (both intervertebral disc extrusion and protrusion) were observed during surgery. If a dog had multiple MRI studies, only the MRI acquired immediately prior to surgery was used.

### Image analysis

MRI studies were randomized, anonymized, and assessed independently by two reviewers (two ECVDI board-certified radiologists). Reviewers were blinded to signalment and diagnosis, reviewing all studies on standard image archiving and communication system software (version 11.0.3, OsiriX Foundation, Geneva, Switzerland). Reviewers were able to manipulate the images as needed. The reviewers had access to a T2W sagittal sequence including the lumbosacral spine, a T2W transverse sequence over the lumbosacral disc space, and if available a STIR dorsal oblique, sagittal or transverse sequence. Reviewers were not aware of the number of dogs in the population that had a protrusion or an extrusion.

Reviewers were asked to complete a questionnaire which guided assessment of a selection of MRI variables based on earlier reported veterinary studies (4, 6). Assessed variables included the degree of compression, presence of displacement of the cauda equina, degree of degeneration of the intervertebral disc, presence of nuclear cleft, morphology of the disc herniation, localization and lateralization of herniated disc material, presence of narrowing of the intervertebral disc space, presence of epidural hemorrhage, presence of vertebral endplate changes, presence of vertebral malalignment, presence of spondylosis deformans, presence of transitional vertebrae, presence of foraminal stenosis and nerve root changes, and changes in the paravertebral musculature.

Reviewers were asked to make a presumptive diagnosis of intervertebral disc extrusion or protrusion, rate their confidence as “confident” or “not confident” in making this diagnosis, and state which series of images they relied most on to make their diagnosis.

**Figure 1:**
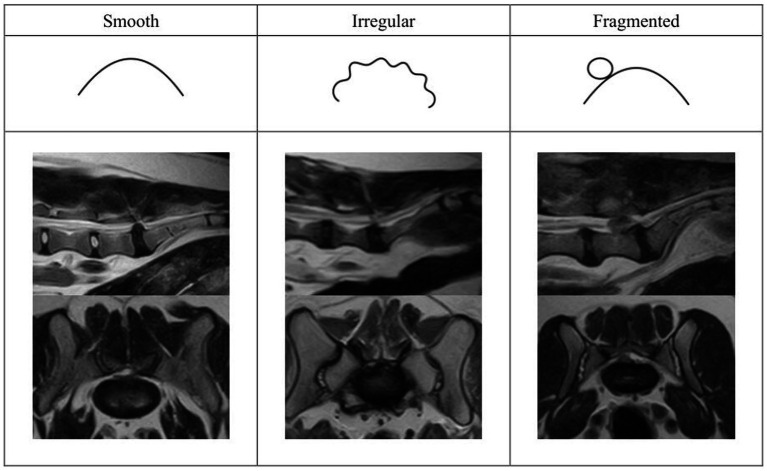
Categories of herniated disc material morphology. From left to right: smooth, irregular, and fragmented.

Degree of spinal cord compression was determined by calculating the percentage of cross-sectional area of the vertebral canal occupied by herniated material at the level of maximal compression. Mild compression was defined as up to 25%, moderate compression as 25–50% and severe compression as >50%. The degree of intervertebral disc degeneration was characterized using the Pfirrmann grading system (13). Nuclear clefts were defined as a focal area of signal loss in the nucleus pulposus on T2W images (14). The morphology of the herniated intervertebral disc was characterized as smooth or irregular if there was smoothly or irregularly marginated extension of the intervertebral disc margin beyond the dorsal border of the vertebral endplate, respectively, (Figures 1A,B). A fragmented appearance was defined as the presence of discontinuity of the herniated material (Figure 1C). Localization of herniated material was assessed in the sagittal and transverse planes. In the sagittal plane, herniated material was described as confined to the intervertebral disc space or dispersed beyond the intervertebral disc space (15). Using both planes, herniated material was categorized as dorsal, ventral, midline and lateralized relative to the spinal cord. The presence of displacement of the cauda equina, narrowing of the intervertebral disc space, vertebral malalignment, vertebral endplate changes, spondylosis deformans, transitional vertebrae, foraminal stenosis and nerve root changes, and changes in the paravertebral musculature were subjectively evaluated on T2W sagittal and transverse images.

### Data analyses

Data were analyzed using statistical software (Prism 8, GraphPad Software, version 8.4.2, 2020, Minitab19 and R 4.2.2). The diagnostic accuracy, sensitivity, specificity, positive predictive value and negative predictive value of MRI for diagnosing intervertebral disc extrusions and protrusions averaged across the two reviewers were calculated using the surgical diagnosis as the reference standard. Cohen’s kappa was used to calculate interobserver agreement and interpretations of these made using published guidelines (16). Association between diagnostic accuracy and reviewer confidence was calculated using Pearson’s chi-squared tests. Associations between the level of reviewer agreement (MRI characteristic not detected by both, equivocal, or MRI characteristic detected by both) and type of intervertebral disc herniation were evaluated using Fisher’s exact tests. Differences in Pfirrmann scores between extrusions and protrusions were examined using Mann Whitney tests. The influence of the choice of image on diagnostic accuracy was determined with a mixed model binary logistic regression (glmer option within R) with choice of image as a fixed effect and dog i.d. as a random effect.

## Results

A total of 117 dogs with surgically confirmed lumbosacral intervertebral disc extrusion (*n* = 16, 14%) or protrusion (101, 86%) were included in this study. The group of dogs with intervertebral disc extrusion included Cocker Spaniels (4), Springer Spaniels (3) and one dog each of the following breeds: Belgian Shepherd Dog, Cavalier King Charles Spaniel, Dachshund, Inuit, Labradoodle, Labrador, Newfoundland, Papillon and Toy Poodle. This group included 11 females (69%) and 5 males (31%) aged between 1 and 10 years (median 6.5 years). The group of dogs with intervertebral disc protrusion included Labradors (34), Cocker Spaniels (9), cross breeds (9), German Shepherd Dogs (7), Dalmatians (4), Labrador Cross (5), Pugs (4), Springer Spaniels (3), Golden Retrievers (3), Cockapoos (2), Jack Russell Terriers (2), and 19 breeds represented by one dog each. This group included 42 females (42%) and 59 (58%) males aged between 2 and 13 years (median 7.1 years). A chi square test between extrusion/protrusion and sex was significant (*χ^2^*(1) = 4.11, *p* = 0.043) suggesting extrusions were more common in female dogs.

### Diagnostic accuracy and interobserver agreement

Overall diagnostic accuracy (Table 1) was 68.8% (95% CI 60.4–77.2%). Cohen’s kappa (based on agreement in diagnosis between reviewers in 82/117 cases) was 0.37; considered fair interobserver agreement. The sensitivity and specificity averaged across reviewers for diagnosing intervertebral disc extrusions at the lumbosacral space was 78.1 and 67.3% respectively, with positive and negative predictive values of 27.5 and 95.1%. Equivalent values for diagnosing protrusions are the complement of the above. For both reviewers, the proportion of false positive diagnosis of intervertebral disc extrusion was greater than false positive diagnosis of intervertebral disc protrusion.

**Table 1:** Overall accuracy of the reviewers.

Reviewer	Total cases	Correct	Incorrect	Accuracy (%)
Reviewer 1	117	78	39	66.7
Reviewer 2	117	83	34	70.9
Mean				68.8

### Diagnostic accuracy and confidence

When making their final diagnosis of extrusion or protrusion, reviewers were asked to subjectively categorize their confidence in making this diagnosis into “confident” or “not confident” categories (Table 2). A “confident” rating was significantly associated with improved diagnostic accuracy (*χ^2^*(1) = 11.66, *p* = 0.001 and *χ^2^*(1) = 18.79, *p* < 0.001 for reviewers 1 and 2 respectively). Averaged across the two reviewers, 85.3% “confident” responses and 52.0% “not confident” responses were accurate (Table 2). There was substantially improved interobserver agreement (κ = 0.89) when both reviewers were “confident” in making their diagnosis. The average accuracy with a confident diagnosis was 86.5% for protrusions and 78.9% for extrusions (Table 3).

**Table 2:** Accuracy of reviewers associated with confidence: **(A)** confident responses and **(B)** not confident responses.

(A)				
Reviewer	Confident cases	Correct	Incorrect	Accuracy (%)
Reviewer 1	53	44	9	83.0
Reviewer 2	64	56	8	87.5
Mean				85.3

(B)				
Reviewer	Not confident cases	Correct	Incorrect	Accuracy (%)
Reviewer 1	64	34	30	53.1
Reviewer 2	53	27	26	50.9
Mean				52.0

**Table 3:** Accuracy in identifying **(A)** intervertebral disc extrusions and **(B)** protrusions, the numbers in brackets refer to the confident + not confident reviews.

(A)			
Extrusion (*n* = 16)	Correct	Incorrect	Accuracy (%)
Reviewer 1	12 (7 + 5)	4 (2 + 2)	75.0
Reviewer 2	13 (8 + 5)	3 (2 + 1)	81.2
Mean			78.1

(B)			
Protrusion (*n* = 101)	Correct	Incorrect	Accuracy (%)
Reviewer 1	66 (37 + 29)	35 (7 + 28)	65.3
Reviewer 2	70 (48 + 22)	31 (6 + 25)	69.3
Mean			67.3

### MRI characteristics significantly associated with intervertebral extrusion or protrusion

**Figure 2:**
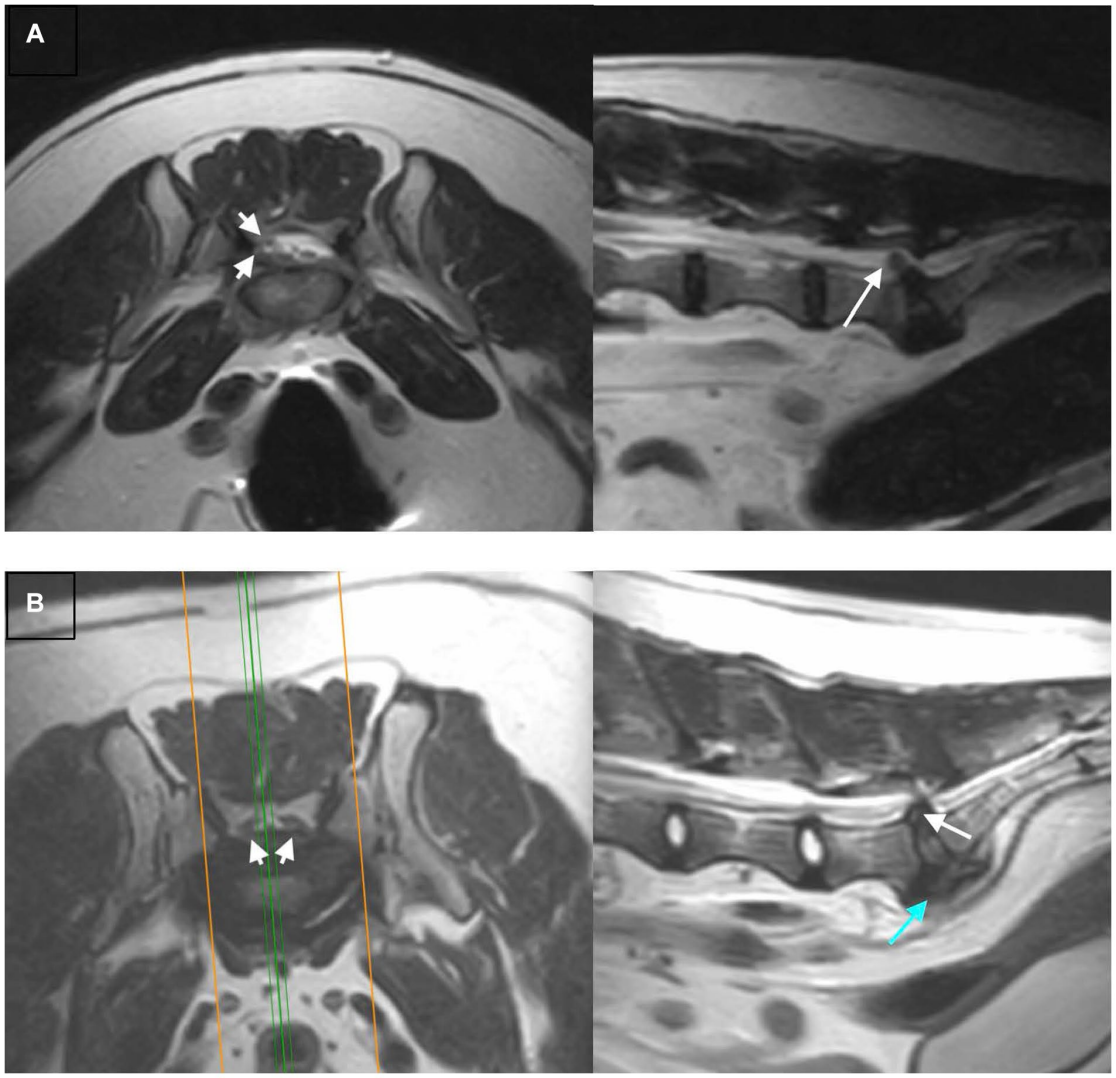
Examples of extrusions vs. protrusions that were correctly identified, T2W transverse (left) and sagittal (right) images are shown for each example: **(A)** An extrusion correctly and confidently identified by both reviewers. There is lateralization of herniated material to the right (arrowheads), and dispersal of the herniated material beyond the margins of the intervertebral disc space (arrow), both features of extrusion. **(B)** A protrusion correctly and confidently identified by both reviewers. The herniated material is midline (arrowheads) and does not extend beyond the margins of the intervertebral disc space (white arrow), both features of a protrusion. The degenerative changes such as ventral spondylosis (blue arrow) are severe.

**Figure 3:**
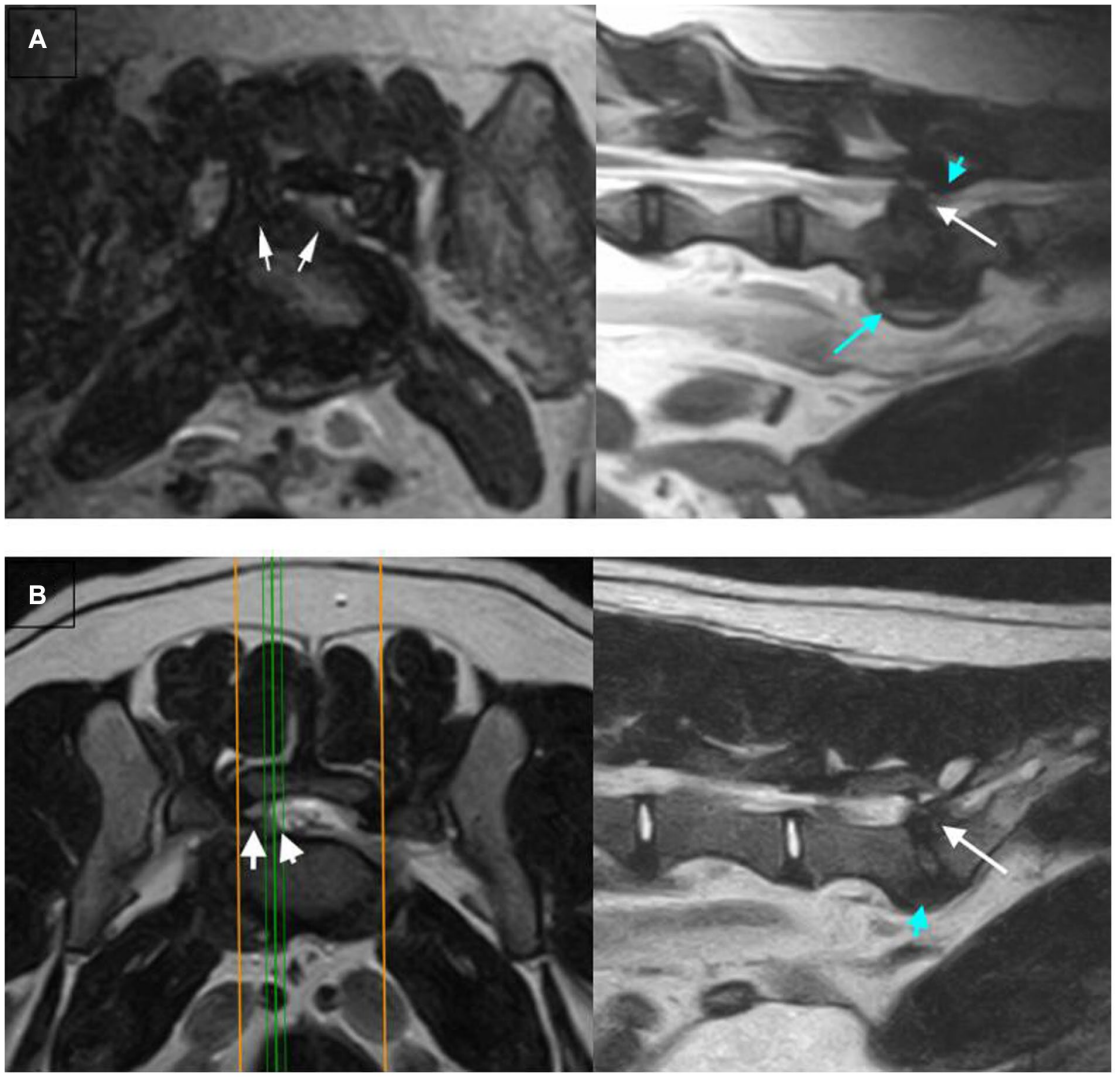
Examples of extrusions vs. protrusions that were incorrectly identified, T2W transverse (left) and sagittal (right) images are shown for each example: **(A)** An extrusion misclassified as a protrusion by both reviewers (unconfident). The transverse image shows the herniated material is lateralized (arrowheads), a feature associated with extrusion. However, features associated with protrusion are identified on the sagittal image – the herniation is confined to the intervertebral disc space (arrow) and dorsal impingement at the lumbosacral disc space (blue arrowhead). The discrepancy of features between the two images has likely led to the unconfident misclassification by the reviewers. **(B)** A protrusion misclassified as an extrusion (confident) by both reviewers. There is lateralization of herniated disc material (arrowheads) with extension of material beyond the margins of the intervertebral disc space (white arrow). These are both features associated with extrusion, likely leading to the reviewers misclassifying this case.

Table 4 summarizes the detected MRI characteristics associated with extrusion or protrusion. Interruption of the dorsal annulus, suspected presence of epidural hemorrhage, dispersed (rather than confined) intervertebral disc on the T2W sagittal, displacement of the roots of the cauda equina, and lateralization (rather than midline) of the herniated material, were significantly associated with intervertebral disc extrusion at the lumbosacral space. Conversely, dorsal impingement at the lumbosacral intervertebral disc space, altered signal intensity of the vertebral endplates, confinement of intervertebral disc herniation to the margins of the lumbosacral disc space and midline intervertebral disc herniation were significantly associated with intervertebral disc protrusion at the lumbosacral space. Regarding the shape of the herniated material, a fragmented appearance (at least two separate fragments of disc material) was only seen in cases of extrusion (10/24 extrusion responses were fragmented, the remainder were classified as irregular). An example of a correctly identified extrusion, and protrusion, showing many typical characteristics, and two incorrectly identified examples are shown in Figures 2, 3.

**Table 4:** Analysis of individual MRI features in their ability to discriminate between extrusion (maximum *n* = 16) or protrusion (maximum *n* = 101).

Feature		Both reviewers negative	Equivocal	Both reviewers positive	*p*-value
Interruption of the dorsal annulus	E	26.7% (4/15)	46.7% (7/15)	26.7% (4/15)	0.027
	P	54.6% (54/99)	38.4% (38/99)	7.1% (7/99)	
Suspected epidural hemorrhage	E	68.8% (11/16)	18.8% (3/16)	12.5% (2/16)	0.034
	P	87.0% (87/100)	12.0% (12/100)	1.0% (1/100)	
Dispersed disc material on the T2 sagittal (*cf.* confined)	E	56.2% (9/16)	6.2% (1/16)	37.5% (6/16)	<0.001
	P	90.9% (90/99)	5.0% (5/99)	4.0% (4/99)	
Displacement of the cauda equina	E	0	0	100.0% (16/16)	0.039
	P	8.0% (8/100)	22.0% (22/100)	70.0% (70/100)	
Lateralized herniation (*cf.* midline)	E	14.3% (2/14)	14.3% (2/14)	71.4% (10/14)	0.043
	P	39.4% (39/99)	25.2% (25/99)	35.4% (35/99)	
Presence of a nuclear cleft	E	81.2% (13/16)	12.5% (2/16)	6.2% (1/16)	0.082
	P	49.5% (50/101)	28.7% (29/101)	21.8% (22/101)	
Presence of lumbosacral spondylosis	E	50.0% (8/16)	18.8% (3/16)	31.2% (5/16)	0.078
	P	27.3% (27/99)	13.1% (13/99)	59.6% (59/99)	
Dorsal impingement	E	87.5% (14/16)	12.5% (2/16)	0	0.025
	P	52.1% (50/96)	29.2% (28/96)	18.8% (18/96)	
Asymmetry of the paravertebral muscles	E	81.2% (13/16)	12.5% (2/16)	6.2% (1/16)	0.156
	P	57.1% (56/98)	34.7% (34/98)	8.2% (8/98)	
Narrowing of the intervertebral disc space	E	80.0% (12/15)	20.0% (3/15)	0	0.548
	P	67.7% (67/99)	21.2% (21/99)	11.1% (11/99)	
Altered signal intensity of the vertebral endplates	E	25.0% (4/16)	50.0% (8/16)	25.0% (4/16)	0.045
	P	34.6% (35/101)	19.8% (20/101)	45.5% (46/101)	

From left to right the columns refer to cases where both reviewers did not identify the feature, where the reviewers were equivocal, and where both reviewers identified the feature. E indicates extrusion and P protrusion.

### MRI characteristics not significantly associated with intervertebral extrusion or protrusion

The presence of narrowing of the intervertebral disc space, foraminal stenosis and/or changes to the L7 nerve roots, the degree of cauda equina compression, the degree of effacement of epidural fat within the vertebral canal and the Pfirrmann grade of the intervertebral disc were not significantly associated with the specific type of intervertebral disc herniation. The median Pfirrmann grading of the lumbosacral intervertebral disc in extrusions and protrusions were 3 and 4, respectively. There was no significant difference in the Pfirrmann grading for extrusions and protrusions (*p* = 0.749 and *p* = 0.250 for reviewers 1 and 2 respectively).

### Preferred MRI series and diagnostic accuracy

Reviewers were asked to select which of the provided series they relied upon most to make their final diagnosis of extrusion or protrusion. On average, the T2W sagittal images were most relied upon in 61.1% and the T2W transverse images in 38.9%. Diagnostic accuracy was significantly higher (76.3% *cf.* 57.1%) when the T2W sagittal images were most relied upon (OR = 2.80, 95% CI = 1.35–5.84, *p* = 0.006). The STIR images were only available in 51 cases. When available, they were only considered helpful in reaching a diagnosis in 11.6% of decisions.

## Discussion

Prognosis and treatment options differ between patients with thoracolumbar intervertebral disc extrusion and protrusion (17), and although there is limited evidence, probably differs with extrusions and protrusions at the lumbosacral intervertebral space. The management strategies for degenerative lumbosacral stenosis have been widely described, and include medical therapy (18) and various surgical treatments such as dorsal laminectomy with or without discectomy, foraminotomy and fixation-fusion (19–22). On the contrary, apart from a case series that documented excellent outcome and resolution of presenting clinical signs in 11/13 dogs with lumbosacral extrusion following dorsal laminectomy (11), there is little discussing the prognosis and treatment outcomes of intervertebral disc extrusion at this site. Consequently, accurate differentiation of intervertebral disc extrusion and protrusion at the lumbosacral disc space is indicated for assessing the necessity of surgical intervention and for surgical planning.

This study evaluated the diagnostic accuracy of low-field MRI in the differentiation of lumbosacral intervertebral disc extrusions and protrusions. Although some of the characteristics evaluated were extrapolated from a previously published classification system (4, 6), the purpose of this study was not to measure the utility of this system as this would have required the reviewers to be blinded to this information when this method of classification has already been engrained in their training.

Overall accuracy and interobserver agreement were however lower compared to previous reports evaluating the accuracy of MRI guidelines for differentiating thoracolumbar intervertebral disc extrusions and protrusions (6). This discrepancy with previous studies may reflect the proportion of herniations that do not conform to the published MRI features. We speculate that the loss of epidural fat at the level of herniation in combination with the commonly observed degenerative changes surrounding the cauda equina made it difficult to distinguish herniated disc material from the dural sac and adjacent nerve roots (7), limiting the usefulness of some features that provide greater diagnostic accuracy. In addition, previous studies evaluating thoracolumbar intervertebral disc herniations utilized a high-field 1.5 Tesla MRI scanner (4, 6). The low field strength of the magnet used in this study and the inherently lower image signal-to-noise ratio and lower spatial resolution of the images provided (23) may also have influenced the diagnostic accuracy. The thicker slices required to overcome the inherently lower image signal would have resulted in greater voxel size and greater partial volume averaging artifact (24). Reviewers would have found distinguishing anatomical structures (such as distinguishing the cauda equina from herniated disc material) and detecting features (such as interruption of the dorsal annulus for the diagnosis of extrusions) more challenging. A wider selection of MRI sequences not available with low-field MRI scanners could also have helped in discriminating structures, such as by improving spatial resolution or taking advantage of the intrinsically different relaxation characteristics of tissues (23). Low-field MRI faces inherent limitations in image quality due to decreased signal and so awareness of these challenges as demonstrated by this study is important for case selection, as well as for managing clinician and client expectations. However, low-field MRI remains a viable option because of its lower cost and smaller device footprints, and it remains relevant in the growth of outpatient neuroimaging (23). Despite the utility of MRI in advancing patient care, high costs and technical barriers associated with high-field devices have meant availability is still limited, particularly in reference to low- and middle-income countries (25).

A significant improvement in accuracy and interobserver agreement was observed when the reviewers were confident. However, the confidence of the radiologists at the time of reporting was generally low and likely reflected the inability to identify the distinguishing features that they normally rely upon. Studies analyzing radiologist performance do not always report on their confidence in making the diagnosis, but this may be a useful feature on the clinic floor where a reviewer’s confidence in their diagnosis of protrusion or extrusion may affect the therapeutic plan and whether further diagnostic steps are pursued. Some of the issues resulting in poorer radiologist confidence could be overcome with high-field MRI images as referred to above.

The purpose of this study was to assess radiologists’ diagnostic accuracy when reviewing low field MRI images of the lumbosacral space. Therefore, reviewers in this study were blinded to the signalment and clinical history of included dogs. Access to this information would likely have resulted in improved diagnostic accuracy and interobserver agreement as patients with intervertebral disc extrusions often present with an acute onset of clinical signs (4, 26). An evaluation of clinical characteristics associated with IVDE and IVDP at the lumbosacral site was beyond the scope of this study which aimed to assess the contribution of MRI to decision making and would anyway have been limited by the retrospective nature of the study and inherent lack of standardized neurological assessment and record keeping. Subsequently, the clinical characteristics of herniations of the thoracolumbar spine and lumbosacral disc space have been described and for now the reader is referred to the current literature (4, 11, 27).

Five MRI characteristics were significantly associated with intervertebral disc extrusion on univariate analysis in this study. In a case series of MRI findings from 13 dogs with lumbosacral intervertebral disc extrusion, the two features most frequently identified were lateralization (13/13) and dispersal of herniated material beyond the boundaries of the intervertebral disc space (10/13) (11). In accordance with this previous study, lateralization of the intervertebral disc herniation and dispersal of herniated disc material relative to the lumbosacral disc space were found to aid in the differentiation of extrusion or protrusion. Additionally, the suspicion of epidural hemorrhage was significantly associated with extrusions in this and previous studies (6, 28). However, it is possible that the changes attributed to potential hemorrhage in this study were not in fact hemorrhage and instead epidural steatitis (29). Steatitis was documented in the aforementioned case series as a feature leading to contrast enhancement of the epidural tissues and was identified at surgery in 1/13 cases (11). Other sequences, such as T2*W, T1W post contrast and other fat suppression sequences, may have been useful for recognizing hemorrhage or steatitis but were not available and were not evaluated in this study.

Consequently, midline herniation and confinement of the herniation to the margins of the lumbosacral intervertebral disc space were significantly associated with protrusion. These are features that have also been described in thoracolumbar intervertebral disc protrusions (4, 6). Dorsal impingement of the cauda equina (such as by the interarcuate ligament or articular facet pathology) which could reflect more chronic degenerative change, was also significantly associated with protrusion. However, other features of chronicity such as spondylosis deformans, presence of nuclear cleft and asymmetry of the paraspinal muscles approached but did not reach statistical significance. The presence of a nuclear cleft is associated with early intervertebral disc degeneration (14, 30) but our findings are similar to those of Kranenburg et al. (31), where the mean Pfirrmann grade was not significantly different between dogs with extrusion and protrusion. The absence of spondylosis deformans and muscle asymmetry in some patients with protrusion could reflect variation in patient clinical presentation, and some patients may have presented earlier before there was more significant disease. We also hypothesize that features associated with chronic degenerative change may be associated with protrusion but would not allow differentiation of acute extrusions on chronic protrusion.

Intervertebral disc protrusion is the most reported type of herniation at the lumbosacral space (5, 7), as part of the multifactorial condition that is degenerative lumbosacral stenosis. This study population confirms that protrusions are more common at the lumbosacral space, comprising 86.3% of our study population compared to the 13.7% of extrusions. This may be another reason for the lower diagnostic accuracy observed in this study, as reviewers were blinded to the final diagnosis and the number of intervertebral disc protrusions vs. extrusions in the study population. Knowledge that the purpose of the study was to differentiate intervertebral disc extrusion from protrusion may have influenced the reviewers, resulting in an overdiagnosis of extrusions. In clinical practice, knowledge of the prevalence of lumbosacral intervertebral disc protrusions would most likely affect the final diagnosis.

Limitations of this study include that this was a retrospective study and therefore imaging acquisition protocols were not standardized. Although all patients were positioned in dorsal recumbency with their hind limbs extended, this was not standardized, and patients may have experienced different degrees of pressure on their hind limbs. This may have altered the morphology of the lumbosacral space (32–34) and affected the degree of cauda equina compression and foraminal stenosis perceived (35–37). Subsequently, only T2W images and occasional STIR dorsal oblique images were included because these were the most consistently acquired series. The absence of T1W, STIR and T2*W sequences may have resulted in reduced sensitivity in the detection of bony changes such as lumbosacral step, the detection and classification of bony changes and the detection of epidural hemorrhage, and may have affected the categorization of vertebral end plate changes (28, 37–39).

Another limitation was that the inclusion criteria meant there was a degree of selection bias, as all patients included in the study had surgically confirmed intervertebral disc extrusion or protrusion. Surgical confirmation was necessary to provide the “gold standard” diagnosis to assess diagnostic accuracy. However, this meant that patients included in the study had clinical findings and/or imaging findings that were considered severe enough to warrant surgical intervention.

Although the population of dogs with intervertebral disc extrusion at the lumbosacral space was small, this disparity between the prevalence of intervertebral disc extrusion and protrusion at the lumbosacral space reflects what is observed in clinical practice. It could be argued that it would be hard to draw any meaningful conclusions from this small population, but the MRI characteristics significantly associated with this population in this study correlated with characteristics described with extrusions at other intervertebral disc spaces (4), and in a previous dual-center case series (11).

Finally, the reviewers in this study comprised only two veterinary radiologists. Agreement was fair between these reviewers, reflecting the subjective nature of several of the MRI characteristics evaluated. Where possible, a grading system was used in alignment with previously published schemes [such as the Pfirrmann grading (13)]. Visual aids, such as in the classification of the morphology of the herniation, were also used where possible. Discrepancies could be related to reviewer experience but evaluating the effect of reviewer training and background, or the effect of a larger cohort of reviewers was beyond the scope of this study.

In conclusion, the results of this study demonstrate that differentiation between intervertebral disc extrusion and protrusion is less accurate in the lumbosacral space than in the thoracolumbar region. This study also confirmed that previously identified MRI variables that identify disc extrusion elsewhere in the spine, including dispersal and lateralization of disc material, and suspected epidural hemorrhage, remain features associated with lumbosacral intervertebral disc extrusion. In cases where the radiologist did not feel confident in the diagnosis, diagnostic accuracy was poor, and was most likely due to inability to identify characteristic features in low-field MRI or overlap of features in some cases. Further studies could be performed to investigate the utility of high-field MRI and other MRI sequences in differentiating the two disorders at the lumbosacral space, as well as describe the relationship between lumbosacral intervertebral disc extrusion with prognosis and treatment options in a larger population.

## Author’s note

Preliminary results from this study were presented in abstract form at the 2020 ECVDI online congress.

## Data availability statement

The original contributions presented in the study are included in the article/supplementary material, further inquiries can be directed to the corresponding author.

## Ethics statement

Ethical approval was not required for the studies involving animals in accordance with the local legislation and institutional requirements because it is a retrospective descriptive study. Written informed consent was not obtained from the owners for the participation of their animals in this study because a written informed consent to retain their animal’s clinical information for use in future clinical studies was signed on hospital admission.

## Author contributions

HS: Writing – original draft, Writing – review & editing. AC: Conceptualization, Supervision, Writing – review & editing. GC: Conceptualization, Writing – review & editing. TS: Data curation, Formal analysis, Writing – review & editing. DH: Supervision, Writing – review & editing.
